# Supplementary figures and images for: Personalized MRI-based characterization of subcortical anomalies in Ataxia-Telangiectasia using deep-learning
Source: PLoS One. 2025 Aug 29;20(8):e0328828. doi: 10.1371/journal.pone.0328828 (PMC12396669; doi:10.1371/journal.pone.0328828)

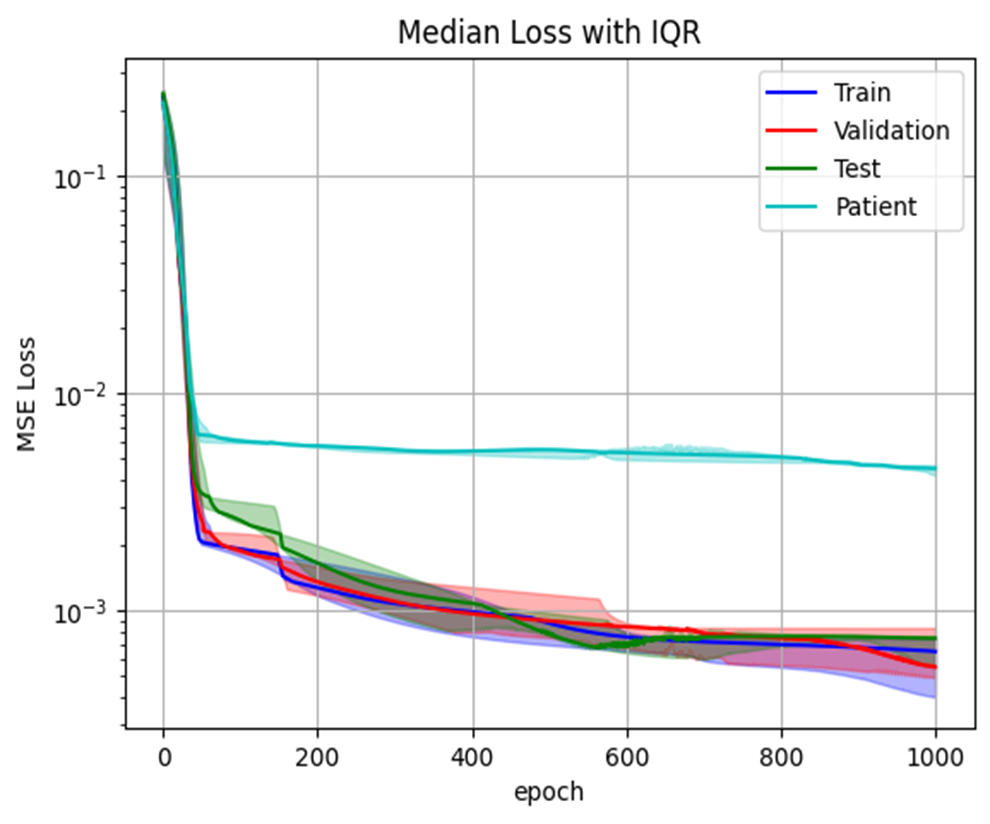

Supplement: S1 Fig — The median MSE illustrates the loss evolution for each group for the five best models and its interquartile range (IQR). (TIF) [file pone.0328828.s001.tif]
