# Supplementary material for: Personalized MRI-based characterization of subcortical anomalies in Ataxia-Telangiectasia using deep-learning
Source: PLoS One. 2025 Aug 29;20(8):e0328828. doi: 10.1371/journal.pone.0328828 (PMC12396669; doi:10.1371/journal.pone.0328828)
Supplement: S1 File — (DOCX) [file pone.0328828.s002.docx]

**Appendix A. Supplementary Information**

Appendix A.1. CATNAP MRI Acquisitions

3D fast spoiled gradient echo T1-weighted structural MRI (1 mm isotropic resolution, TR = 8.15 ms, TE = 3.172 ms, TI = 900 ms, matrix size = 256×256, 156 sagittal slices). EPI-based axial diffusion-weighted imaging (participants were scanned with at least one of two sequences, a more extended protocol for those tolerating the scan well:  TR = 8,000 ms, TE = 63 ms, b = 1,000 s/mm^2^, 32 separate non-orthogonal directions, four additional b = 0 s/mm^2^  images acquired, 2 mm isotropic voxel size, whole brain coverage, number of slices = 66; or shorter protocol if not tolerating the scan well: TR = 8,000 ms, TE  = 83 ms, b = 1,000 s/mm^2^, three orthogonal directions, 0.9 mm × 0.9 mm × 4 mm voxel size, number of averages = 2, whole brain coverage, number of slices = 30).

Pseudo-continuous Arterial Spin Labelling (pCASL) sequence with 3D spiral read-out (tag/control image pairs = 72, flip angle = 111°, TE = 10.536 ms, TR = 4,844 ms, labelling duration = 1,450 ms, post labelling duration = 2,025 ms, field of view = 240 mm, slice thickness = 4 mm, slice gap = 4 mm, number of slices = 36, echo train length = 1, number of excitations = 3, matrix = 128×128, and voxel resolution = 1.875×1.875 ×4 mm).

Appendix A.2. Normative Modelling

To find the optimal hyperparameters, we used grid search to tune the learning rate and select the set of five models (one for each cross-validation fold) with the lowest median validation loss.  We then reconstructed MRI images by taking the median of the outputs from these five models (1, 000 epochs; batch size = 16; LR grid = 10^–2^, 5×10^–3^, 10^–3^, 5×10^–4^, 10^–4^; Adam optimizer; mean square error (MSE) loss) (**Supplementary Fig. 1**).


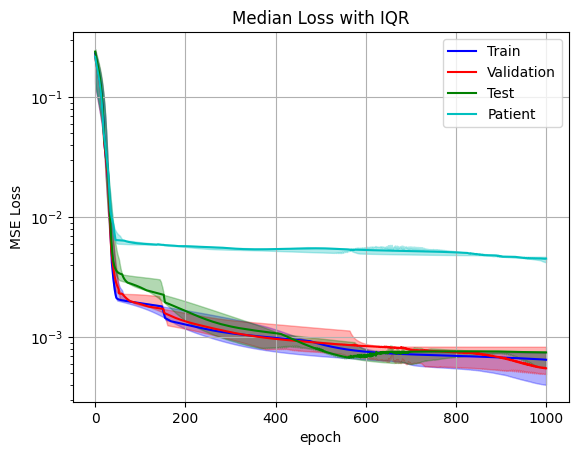


**Supplementary Figure 1.** Median Mean Squared Error (MSE) loss comparison among the five best model sets for training (80 controls), validation (20 controls), test (10 controls), and A-T set (16 participants with A-T). The median MSE illustrates the loss evolution for each group for the five best models and its interquartile range (IQR).
