# Supplementary material for: Personalized MRI-based characterization of subcortical anomalies in Ataxia-Telangiectasia using deep-learning
Source: PLoS One. 2025 Aug 29;20(8):e0328828. doi: 10.1371/journal.pone.0328828 (PMC12396669; doi:10.1371/journal.pone.0328828)
Supplement: Attachment — Submitted filename: Response to Reviewers.docx [file pone.0328828.s004.docx]

We would like to thank the two reviewers for their kind and insightful comments. We are sure that by addressing them we have strengthen and improved our manuscript substantially.

**Comments to the Author**

Reviewer #1: The authors utilize a novel method, a normative self-supervised deep autoencoder, which they trained on MRI diffusion and perfusion sequences from healthy controls. They then apply this trained model to a set of patients with ataxia-telangiectasia and identify regions where reconstruction errors occurred, highlighting regions of brain pathology in this disease. Before reading this submission, I was not aware of this methodology, and the writing is clear and the reasoning well explained in the manuscript. The findings are well described, as are the figures. Moreover, the discussion is clear and highlights the limitations, namely that the directionality of the findings is unknown. Moreover, no pathophysiologic target can be identified using these methods, only a brain region that is affected. Overall, I have no editorial suggestions and think this paper is worthy of publication with little or no additional editing.

Authors’ response: Thank you very much for your very kind review.

Reviewer #2: The study presents an investigation into the basal ganglia (motor circuits) in Ataxia-Telangiectasia (A-T) patients, utilizing a normative self-supervised deep autoencoder trained to highlight deviations in imaging features (in this case perfusion and diffusion) compared to healthy controls. The results are positioned as exploratory and may serve as a foundation for future in-depth investigations. Below are specific comments regarding different sections of the manuscript.

Introduction
Q1. - The motivation for investigating perfusion and diffusion differences in A-T patients could be better articulated. Are there specific biological mechanisms or prior findings that suggest these parameters would be affected? Providing a clearer rationale would strengthen the introduction.

Authors’ response: Thank you very much for this comment. We have provided more details of this in the introduction with the following text:

*“Previous animal models of A-T have shown that nigrostriatal degeneration is detectable in ATM-deficient mice [10,11]. We postulated that ADC may provide an index of ultrastructural changes in the basal ganglia reflecting degeneration in A-T. We also postulated that perfusion changes may occur in the basal ganglia following prior work showing altered basal ganglia metabolism [12], on the basis that blood perfusion and metabolic activity are coupled.*”

Methods
Q2. - The manuscript states that only imaging features are reconstructed using the model. Additional details on how covariates (e.g., age, sex) are incorporated or accounted for within the model would help clarify their impact on model performance.

Authors’ response: Thank you for this helpful suggestion. To clarify how covariates are incorporated into the model, we have revised the Results section to explicitly describe the final architecture. While demographic covariates are part of the input to inform encoding, the model is explicitly trained to reconstruct only the imaging features. This design allows the autoencoder to account for potential demographic effects on imaging patterns. We now state in the caption of Figure 2A (in Results 2.4)):

*“Schematic of the autoencoder architecture and overall research design. The autoencoder comprises seven asymmetrical, fully connected layers. The input layer includes 20 features: six perfusion features (green: caudate, hippocampus, pallidum, putamen, thalamus, cortical gray matter (CGM)), seven diffusion features (orange: hippocampus, pallidum, putamen, thalamus, CGM, cerebral white matter (CWM)),and seven covariates (purple: age, sex, cerebral volume, cerebellar volume, age², age × sex, age² × sex). The output layer contains 13 features, enabling reconstruction of only the six perfusion (green) and seven diffusion (orange) features.*”

In order to understand the impact of the covariate features in the reconstruction, we analysed the weights learned by the neural network that directly connected to those seven covariate inputs. As expected, the connection weights of input covariates to the first hidden layer were smaller (M(w_ij_)=0.068, SD=0.0469) than the weights associated to the 13 input imaging features (M(w_ij_)=0.1107, SD=0.0858), but they were not negligible. Thus, the impact of covariates was deemed relevant for image reconstruction in the AE model after analysing their direct effect on the first hidden layer, something that can also be observed by inspection of the weight matrix (Attached fig. 1).

Attached figure 1. Each row represents an input feature (20 in total), and each column corresponds to a neuron in the first hidden layer (15 in total). Cell colours indicate the weight of the connection between each input and neuron (w_ij_). The top six rows represent perfusion variables, the middle seven correspond to diffusion variables, and the bottom seven rows represent covariates.

The summarized weights magnitudes were also in the same order of magnitude as imaging features, and thus non negligible (Attached table 1). The distribution is further illustrated using boxplots.

| **Input group** | **Mean \|w_ij_\|** | **SD** |
| --- | --- | --- |
| Imaging features (top 13) | 0.1107 | 0.0858 |
| Perf. Imaging (top 6) | 0.1431 | 0.0980 |
| Diff. Imaging (middle 7) | 0.0830 | 0.0613 |
| Covariates (bottom 7) | 0.0680 | 0.0469 |

Attached table 1. Mean and standard deviation of absolute weights of the connection between input features and neurons in the first hidden layer. Rows show the different types of input features: imaging features (diffusion and perfusion features, both aggregated and separated), and covariates.

Attached figure 2. Boxplots for weight distribution of input features on first hidden layer, for perfusion, diffusion and covariate features (Demo = Demographic covariate features).

We think this analysis is important for clarification, but it is not directly related to the article’s main contribution, so we just provide this information here.

Q3. - It is unclear whether the state of participants during scanning (awake vs. asleep) was documented. Brain perfusion can vary significantly based on the state of consciousness (and therefore potentially between controls and patients), and this factor should ideally be addressed in the methods section.

Authors’ response: Thank you very much for pointing this out. We completely agree with the reviewer that effects in perfusion due to state of consciousness is an important factor to consider. For this reason, we contacted the researchers from Calgary, and they were very helpful in providing us with information about alertness/sleepiness during scans (all participants from Nottingham, which includes all A-T participants, were awake during scanning). Turns out that from the total of 110 controls we used (from both Nottingham and Calgary), only 6 were asleep during scanning. For this reason, we argue that in the particular case of this study, the effect will be negligible. We have added the following text about this in the limitations section of the discussion:

*“Another limitation of the present study is that although state of consciousness (awake vs sleep) can have an impact on brain perfusion (46), we did not take this into account because none of the A-T participants and only 6 of the 110 controls subjects that were considered were sleeping during scanning.”*

Results/figures
Q4. - Further information is needed on how age, sex, and cerebral volume influenced the correlation between reconstruction errors (this is related to my earlier point on the inclusion of covariates in the model) and clinical scores. These factors could have significant effects on the findings and should be reported.

Authors’ response: In this version, we employed LASSO regression to analyze pairwise correlations between clinical scores and reconstruction errors of imaging features. To account for a priori defined biological relevance, covariates including age, age², sex, sex × age, sex × age², cerebral volume, and cerebellar volume were included in the model as unpenalized terms. This approach preserves their contribution while allowing LASSO to select among the imaging features. These details are now clearly stated in the Methods section 2.5 and the results reported in section 4.5.

Q5. - The interpretation of differences between controls and A-T patients based on the UMAP representations should be elaborated upon. What insights could these representations provide regarding differences between patients and controls in terms of features?

In our UMAP analysis, we observed that the latent representations (the central layer of the autoencoder) derived from perfusion and diffusion features effectively distinguish between control and A-T groups, indicating that these imaging features contain informative patterns relevant to group separation.

To better interpret these differences, we conducted complementary analyses, including SHAP value inspection and feature importance estimation via LASSO regression (see Sections 4.4 and 4.5). These analyses highlight how individual imaging features contribute to model predictions and demonstrate that several features are associated with clinically relevant abnormalities in the A-T group. A detailed list of specific imaging feature differences between A-T and control participants is provided in Section 4.3.

As a sensitivity analysis, we trained an additional model with a reduced architecture which considered only imaging input features (input layer: 13 nodes; hidden layers: 11, 8, 4, 8, 11; output layer: 13 nodes) and compared it to our original “extended” model, which includes both imaging and covariate inputs. Both models were trained using identical procedures and achieved comparable median test MSEs (0.00055 for the extended model, 0.00064 for the reduced model; Wilcoxon p-value p = 0.53).

However, only the extended model produced latent UMAP representations that clearly separated control from A-T subjects (see Figure 3, test group only; Figure 4, entire dataset). This supports our interpretation that the inclusion of covariates enhances latent feature separation and further validates the role of imaging and covariate interactions in distinguishing A-T from control participants.

Attached figure 3. UMAPs for the original (extended model), and the alternative reduced model in consideration, including test control and A-T participants, coloured by participant status. If possible, we included a participant status separation line.

**UMAP – Extended Model	UMAP – Reduced Model**

Attached figure 4. UMAPs for the original model, including the complete control set coloured by participant status.

While we believe this analysis offers useful clarification, it is not central to the article’s main contribution, so we just provide this information here.

Q6. - Figure 3 would benefit from a clear color legend or bar to enhance interpretability.

Authors’ response: Thank you for letting us now. Figure 3 includes colour legend now.

- The x-axis label of figure 4 is confusing. As these are stacked bar plots, should this not be the sum of the means instead of (just) mean?

Authors’ response: Thank you for this comment. We have addressed this and changed the current label.

Discussion
Q7. - The use of SHAP analysis is an interesting approach to better understand the model output; however, it remains somewhat unclear how results from different structures could influence each other. Additional explanation on how these changes relate to the brain’s subcortical motor circuits would be helpful.

Authors’ response: Thank you for this comment. We have further expanded/reworded the discussion to explain these relationships with the following paragraphs:

*“The SHAP analysis showing that perfusion or diffusion values of cerebellar cortical or supratentorial sub-cortical gray matter structures can be ‘explained’ to differing extents by the perfusion or diffusion values in others of these structures suggests a network-level effect of A-T. Indeed, as well as the well-established classical motor control circuits involving the basal ganglia and thalamus, recent evidence also demonstrates the presence of direct cerebellar-basal ganglia and cerebellar-thalamic circuits [44]. These provide a potential biological substrate for disease-related network-level alterations in diffusion (reflecting tissue ultrastructure) and metabolically coupled perfusion parameters.*

*However, it is important to recognize the fundamental limitation of the SHAP explainability analysis which is the uncertainty in determining the direction of the observed correlations. While associations are evident, this analysis does not reveal causality. Thus, it is unclear which features cause changes in others, raising the possibility that the relationships observed may be due to confounding variables rather than actual causal effects. For example, we note that the hippocampus, included in the analysis to provide a non-motor supratentorial subcortical structure, was found to be an explanatory feature for diffusion and perfusion alterations in other subcortical structures participating in motor control circuits. This was an unexpected and possible spurious finding or could potentially relate to the recently described interplay between the cerebellum and hippocampus [45]. The limitation of the SHAP explainability analysis emphasizes the need for caution in interpreting the results. Future research could employ methods such as functional analysis, time series analysis, and longitudinal studies to better understand the underlying causal mechanisms.”*

Q8. - The SHAP analyses further suggests that input features contributed equally (positively or negatively) in most cases to the output features, except for cerebellar gray matter diffusion (for perfusion output) and caudate and putamen perfusion (for diffusion output). This is also reported in section 4.4 of the results, but is not elaborated further on in the discussion. This observation warrants further discussion.

Authors’ response: Thank you for your comment. We agree with the reviewer that this warrants further clarification. Despite this, we see that the extent of SHAP contributions for these features that are both positive for some and negative for others tend to be quite modest and likely spurious / negligible, especially given the modest sample size. For example, for diffusion output we have that the SHAP values of caudate perfusion as input are in the order of 10 x 10^-6^ mm^2^/s or less, which is just a small proportion of normal parenchymal ADC values that range around [600 - 1000] x 10^-6^ mm^2^/s. Considering this, we have added some insights about these results in the section where it is reported (section 4.4) with the following text:

*“For both perfusion and diffusion output features, the direction of the relationships shown for each of the individual input features (indicated by either positive or negative aggregated mean SHAP values) was largely consistent. However, several exceptions to this were noted. For example, for the CGM diffusion input, both the caudate and CGM perfusion outputs had negative mean SHAP values, whereas the other perfusion outputs had positive mean SHAP values. Similarly, both the caudate diffusion and putamen diffusion inputs showed positive and negative mean SHAP values for the relevant diffusion outputs. While speculative explanations could be proposed whereby disease-related loss of either excitatory or inhibitory actions of input structures differentially affects the outputs, in view of the small sample size we suspect these inconsistencies in the direction of the mean SHAP values are likely to be spurious.”*

Q9. - Finally, the differences between positive and negative SHAP values should be explained further. Can these differences be interpreted as their impact to be increasing or decreasing the perfusion/diffusion estimates, respectively? A clearer interpretation would strengthen the discussion.

Authors’ response:

Thanks for this comment. Indeed, the SHAP absolute value of an input variable indicates how much that variable influences the output, and its direction indicates whether an increase of the input feature increases (positive SHAP) or decreases (negative SHAP) the output. Now, the SHAP score must the interpreted in the context of the distribution of the output variable. If the output is a probability, then SHAP values between 0.5-1 can reflect a large impact, but if the distribution of the output variables ranges between 0-100, then a SHAP value of 5 can be deemed to be non-significant. We reflect this explanation in section 2.4 to better interpret our results with the following text:

*“The absolute SHAP value of an input variable indicates how much that variable influences the output, and its direction indicates whether an increase of the input feature increases (positive SHAP value) or decreases (negative SHAP values) the output. However, the SHAP value must the interpreted in the context of the distribution of the output variable. If the output is a probability value, then SHAP values between 0.5-1 can reflect a large impact, but if the distribution of the output variables ranges between 0 and 100, then a SHAP value of 5 can be deemed to be non-relevant.”*

Further stressing SHAP values importance, we added in Discussion, paragraph 6:

*“In A-T patients, SHAP analysis reveals how input features drive anomalies in reconstructed outputs, with contributions interpreted relative to the scales of perfusion (0–100 mL/100 g/min) and diffusion (0–3,000 ×10⁻⁶ mm²/s) and reconstruction errors (1–5 mL/100 g/min for perfusion, tens to hundreds ×10⁻⁶ mm²/s for diffusion). For example, a negative SHAP value for caudate on putamen perfusion indicates that elevated caudate perfusion in A-T corresponds to a reduced putamen perfusion estimate compared to controls, suggesting disrupted inter-regional dynamics. This feature-specific insight refines our understanding of the anomalies observed, complementing broader network-level findings. "*
